# Syndromic Diagnostics for Travelers’ Diarrhea: Near-Patient Field-Expedient Testing in Resource-Limited Settings

**DOI:** 10.1093/ofid/ofag076

**Published:** 2026-02-17

**Authors:** Romeo Toriro, Christopher T Williams, Dominic L Wooding, Thomas Edwards, Matthew K O’Shea, Thomas E Fletcher, Nicholas J Beeching, Daniel S Burns, Stephen D Woolley

**Affiliations:** Academic Department of Military Medicine, Royal Centre for Defence Medicine, Queen Elizabeth Hospital Birmingham,Edgbaston, Birmingham, United Kingdom; Department of Clinical Sciences, Liverpool School of Tropical Medicine, Pembroke Place, Liverpool, Merseyside, United Kingdom; Centre for Drugs and Diagnostics, Liverpool School of Tropical Medicine, Pembroke Place, Liverpool, Merseyside, United Kingdom; Centre for Drugs and Diagnostics, Liverpool School of Tropical Medicine, Pembroke Place, Liverpool, Merseyside, United Kingdom; Centre for Drugs and Diagnostics, Liverpool School of Tropical Medicine, Pembroke Place, Liverpool, Merseyside, United Kingdom; Academic Department of Military Medicine, Royal Centre for Defence Medicine, Queen Elizabeth Hospital Birmingham,Edgbaston, Birmingham, United Kingdom; Centre of Defence Pathology, Royal Centre for Defence Medicine, Queen Elizabeth Hospital Birmingham,Edgbaston, Birmingham, United Kingdom; Institute of Immunology and Immunotherapy, College of Medical & Dental Sciences, University of Birmingham, Edgbaston, Birmingham, United Kingdom; Department of Clinical Sciences, Liverpool School of Tropical Medicine, Pembroke Place, Liverpool, Merseyside, United Kingdom; Department of Clinical Sciences, Liverpool School of Tropical Medicine, Pembroke Place, Liverpool, Merseyside, United Kingdom; Academic Department of Military Medicine, Royal Centre for Defence Medicine, Queen Elizabeth Hospital Birmingham,Edgbaston, Birmingham, United Kingdom; Academic Department of Military Medicine, Royal Centre for Defence Medicine, Queen Elizabeth Hospital Birmingham,Edgbaston, Birmingham, United Kingdom; Department of Clinical Sciences, Liverpool School of Tropical Medicine, Pembroke Place, Liverpool, Merseyside, United Kingdom; Centre of Defence Pathology, Royal Centre for Defence Medicine, Queen Elizabeth Hospital Birmingham,Edgbaston, Birmingham, United Kingdom

## Abstract

**Background:**

We assessed the diagnostic agreement of BioFire FilmArray multiplex polymerase chain reaction (PCR) with Seegene Allplex PCR for testing fecal samples collected during a diarrhea outbreak in resource-limited settings.

**Methods:**

Fecal samples from consented British military personnel training in Kenya were collected without preservative and tested onsite with the FilmArray PCR platform. Anonymized corresponding samples frozen near the point of care were tested 16–18 months later in the United Kingdom using Seegene PCR (reference standard). We compared test sensitivity and specificity and assessed agreement using Cohen κ coefficients.

**Results:**

Samples were analyzed from 60 individuals (80% male; median age [interquartile range], 24 [22–28] years). The overall pathogen detection rates did not differ significantly between FilmArray and Seegene PCR (55 of 60 [91.7%] vs 53 of 59 [89.8%], respectively [*P* > .9]). *Campylobacter* spp detection was significantly higher with Seegene (17 of 59 [28.8%] vs 6 of 60 [10%] for FilmArray PCR *P* = .03). The sensitivity of FilmArray PCR was moderate for *Cryptosporidium* spp (65% [95% confidence interval, 45.37%–80.77%]), and low for *Campylobacter* spp (35.3% [14.21%–61.67%%) and norovirus (7.14% [.18%–33.87%]). Its specificity was good to excellent for detection of *Campylobacter* spp, *Cryptosporidium* spp, enteroaggregative *Escherichia coli,* and sapovirus.

**Conclusions:**

The study shows moderate concordance of FilmArray with Seegene PCR in the detection of 5 enteropathogens and poor to fair concordance for 7 others, but high-quality case-control studies are needed to assess agreement between these platforms. However, based on performance characteristics, including platform versatility and ease of use, and in the absence of a gold (reference) standard test, the FilmArray platform remains a suitable near-patient field-expedient platform for diarrhea diagnostics in resource-limited settings.

Travelers' diarrhea (TD) is a common medical presentation that can have a significant impact on military operational effectiveness in both training and deployment settings [1, 2]. Its etiology depends on a number of factors, including seasonality, travel duration and destination, individual health status, and other determinants of health [3]. During TD outbreaks in resource-limited settings, the need to identify disease etiology is critical in informing clinical case management to curtail disease spread and minimize morbidity and medical evacuation [4] as well as in reinforcing force health protection measures, which include long-term surveillance, as has been highlighted in recent military deployments [1, 3, 5–7]. Rapid near-patient field-expedient (NP-FE) diagnostic capability to identify the causes of TD in forward military deployment locations has historically not been well documented [8]. However, recent studies have reported the utility of culture-independent molecular platforms, such as the field-expedient FilmArray multiplex polymerase chain reaction (PCR) gastrointestinal (GI) panel (bioMérieux) (FilmArray) to provide rapid identification of multiple enteropathogens [9, 10]. Other military studies conducted in austere settings have also highlighted the utility of the FilmArray platform as an ideal alternative or an adjunct to traditional culture methods [4, 6].

The FilmArray PCR platform commissioned for use by the UK Armed Forces in deployed hospital settings targets 22 enteropathogens in a single cartridge-based test on a random-access system with the capability to reduce hands-on time to about 3 minutes [11] and testing turnaround time to <2 hours [12]. The fecal samples are held before testing in Cary-Blair transport medium. The system houses all the chemistry required to extract, amplify, and detect nucleic acid from multiple enteropathogens within a single fecal sample, which can either be held at room temperature at 15°C–25°C or refrigerated at 2°C–8°C, in both cases for up to 4 days. FilmArray software automatically analyzes and interprets the assay results, displaying them qualitatively in a test report [11].

The Seegene Allplex (Seegene) platform is a multiplex 1-step real-time -PCR platform with viral, parasitic, and 2 bacterial assays [13]. These assays allow simultaneous detection of up to 7 enteropathogens within a single reaction tube using a specific detection algorithm, enabling a total of 25 pathogens to be tested across the panel. It is based on multiple genetic quantification by RT-PCR and specific interpretation software, with the panels running in either a combined or a selected manner [13]. The Seegene platform has a run time of <2 hours 30 minutes in a single reaction and has good concordance with other multiplex molecular assays [13–15], including a user-friendly workflow that displays test results as individual cycle threshold (Ct) values of multiple analytes in a single channel on the RT-PCR instrument [16].

Compared with conventional techniques, syndromic testing with platforms such as the Seegene has been described not only as improving and accelerating microbiological diagnosis but also as efficient and cost-effective for patient care [13, 17]. However, in some cases enteropathogens detected may be of uncertain clinical significance, meaning that test results need careful interpretation [10]. Although widespread use of the Seegene platform has led to a paradigm shift in the diagnosis of TD and other infectious diseases in clinical microbiology settings [17], it is less suitable for field diagnostics as it is designed for laboratory use. However, it remains a good comparator to evaluate the performance of the FilmArray platform in TD diagnostics, and it targets more enteropathogens than the FilmArray **(**Supplementary Data Sheets 1 and 2).

Knowledge about the suitability of the FilmArray platform for accurately identifying the causes of TD in forward deployment locations is currently limited. We evaluated the (1) sensitivity and specificity, (2) agreement, and (3) interrater agreement between FilmArray PCR as an NP-FE test and the Seegene platform as the reference standard test for TD diagnostics in resource-limited settings.

## METHODS

### Study Design and Specimen Collection

**Figure 1.**
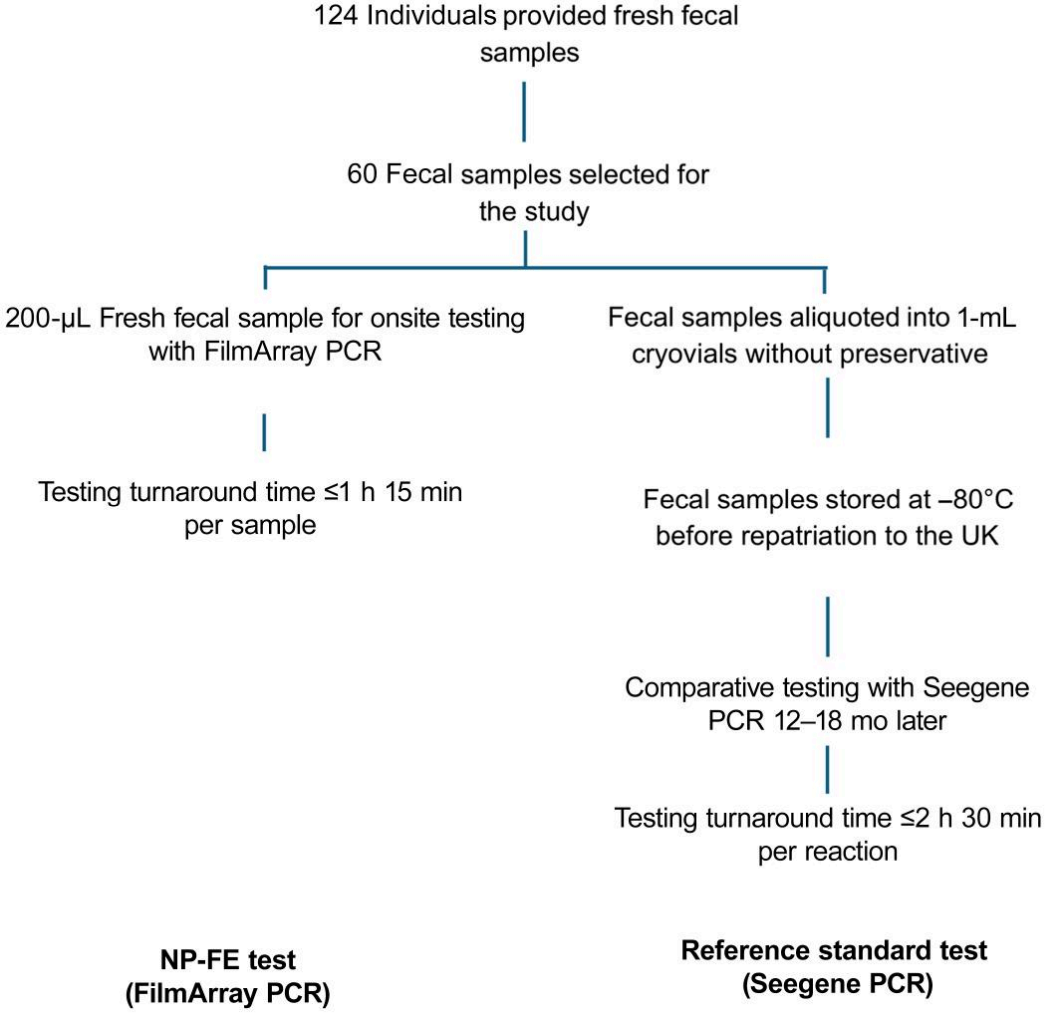
Near-patient field-expedient (NP-FE) and reference standard processing steps. Fecal samples were tested near the point of care for immediate case management, and corresponding samples were repatriated in the cold chain for later comparative testing in the United Kingdom. Abbreviation: PCR, polymerase chain reaction.

Fresh fecal samples from British military personnel who experienced diarrhea during a *Cryptosporidium* outbreak described elsewhere were analyzed using the FilmArray PCR platform as a clinical NP-FE test [6]. Individuals self-collected loose and semisolid stool samples in 500-mL snap-on lid specimen collection pots, before transferring approximately 10-mL stool volumes into 30-mL sterile bottles. Due to resource and time constraints, we selected 60 of 124 samples comprising a mixture of negative samples and single-, dual-, and multiple-enteropathogen samples as detected by FilmArray PCR for comparison with the Seegene platform as the reference standard test. One corresponding Seegene test was invalid and excluded from the analysis. We used quota sampling to select a varied pool of samples, as described elsewhere [18]: negative samples, 5 of 60 (8.3%); single enteropathogen, 19 of 60 (31.7%); 2 enteropathogens, 20 of 60 (33.4%); and multiple enteropathogens, 16 of 60 (26.7%). Corresponding samples from the selection were anonymized, marked with unique identifiers, and aliquoted into 2-mL cryovials without preservative before storage at −80°C. They were then repatriated to the United Kingdom in dry ice at −50°C to −85°C and kept frozen at −80°C before subsequent Seegene testing 16–18 months later (Figure 1) by investigators blinded to the FilmArray test results. We calculated sensitivity and specificity, the overall detection rate (ODR) as TP/(TP + FP), where TP and FP represent true-positive and false-positive, and the percentage observed agreement as TP + TN/(TP + FN + FP + TN) × 100, where TN and FN represent true-negative and false-negative.

### Statistical Analysis and Ethics

The Ministry of Defence Research Ethics Committee ethical approval (no. 2076/MODREC/21) was granted in 2021, and all participants provided informed written consent. Cohen κ statistical coefficients (Microsoft Excel, version 2401) were used to measure the interrater agreement and degree of accuracy between tests using the following formula: κ = (*p*_o_ − *p*_e_)/(1 − *p*_e_), where *p*_o_ represents the relative observed agreement among raters and *p*_e_, the expected hypothetical probability of chance agreement.

### Rationale for Reference Standard Test Choice

In the absence of a known reference or gold standard multiplex PCR platform for GI molecular diagnostics, we selected the Seegene platform as the reference standard test. It has reliable detection capability, good concordance with other multiplex PCRs (depending on the target enteropathogens), including when compared with the FilmArray platform [19], and superior sensitivity of 89%–100%, particularly for common bacterial targets compared with traditional reference standards, including culture and sensitivity [13]. Its ability to detect multiple pathogens in a single channel with individual Ct values and a rapid turnaround provides a degree of objectivity in the quantitative detail that is missing in other platforms like the FilmArray [20] (Supplementary Data Sheets 1 and 2). The FilmArray platform is preprogrammed to generate results qualitatively, whereas the Seegene platform produces both a qualitative summary and quantitative analysis of results. This can inform clinical decisions on whether enteropathogens are commensal or pathogenic, confirming this platform as a suitable comparator.

### FilmArray PCR Testing

Individual FilmArray pouches for each respective sample were placed into the loading block, and the hydration solution was injected into the pouch through the blue inlet port. For each respective sample, approximately 200 µL of stool was added into the sample buffer, and the solution was mixed then injected into the pouch through the red inlet port. Next, the FilmArray pouch was loaded into the instrument, and the pouch identification number scanned using the barcode reader. Finally, the unique respective sample identifiers were entered, and the automated run was started. The test completes with the software assigning test results qualitatively for each of 22 GI enteropathogens, then producing a test report (bioMérieux) (Supplementary Data Sheet 3).

### PCR Testing by Seegene Allplex GI Panel

Nucleic acids were extracted from stool samples using the QIAamp 96 PowerFecal QIAcube HT Kit on a QIAcube HT platform, following the manufacturer's instructions. Initial homogenization of the stool samples was conducted on the QIAcube HT platform. Briefly, 200 mg of each stool sample was placed into a well of a PowerBead Pro plate containing preloaded ceramic beads, and 650 µL of prewarmed PW1 buffer was added. The plate was vortexed continuously for 10 minutes and then centrifuged to pellet the debris (Qiagen). (Supplementary Data Sheet 4).

PCR assays were analyzed on a CFX96 (BioRad) platform, and the results were interpreted using Seegene Viewer software, which automatically interprets positivity and produces a test report, including Ct values (Mast Group, UK).

## RESULTS

### FilmArray Versus Seegene ODRs

Samples from 60 of 124 individuals (48.4%; 80% male; median age [interquartile range], 24 [22–28] years) tested with FilmArray PCR were selected for the study and compared with corresponding samples tested with Seegene PCR, apart from 1 invalid Seegene test that was excluded from the analysis. The ODRs of all enteropathogens were comparable between the FilmArray and Seegene platforms (55 of 60 [91.7%] vs 53 of 59 [89.8%], respectively; *P* > .93). Both platforms detected more bacterial than protozoal or viral enteropathogens, and the ODRs for bacteria were comparable between FilmArray (42 of 60; [70%]) and Seegene (41 of 59 [69.5%]; *P* < .05). The FilmArray platform detected fewer viral enteropathogens than the Seegene (in 6 of 60 samples [10%] vs 20 of 59 [33.9%], respectively; *P* < .02) and fewer protozoal enteropathogens (27 of 60 samples [45%] vs 33 of 59 [55.9%]; *P* < .05). Three of 5 samples negative with FilmArray PCR were positive with Seegene (2 for multiple enteropathogens and 1 for *Dientameba fragilis*). *D fragilis* and *Blastocystis hominis* are not included in the FilmArray panel but were present in 8 of 59 (13.6%) and 30 of 59 (50.8%) Seegene tests, respectively. The FilmArray platform also detected 1 each of *Salmonella* spp and *Entameba histolytica,* while none of either were detected with the Seegene platform.

The Seegene platform detected more *Clostridioides difficile* and norovirus than the FilmArray (in 6 vs 2 samples and 14 vs 1, respectively). Similarly, the detection rate for *Campylobacter* spp was significantly higher with Seegene than with FilmArray PCR (6 of 60 [10%] vs 17 of 59 [28.8%]; *P* = .03). Norovirus and rotavirus were almost always detected in combination in the multiple-enteropathogen samples with Seegene testing. There were only 6 exact matches by the 2 platforms, and 25 of 60 samples (41.7%) were *Cryptosporidium* spp positive. Of these 25 samples, 20 (80%) were also positive with Seegene PCR, but in combination with multiple other enteropathogens in most samples. The Seegene platform detected multiple pathogens in 32 of 59 samples tested (54%). Overall, diarrheagenic *Escherichia coli* (DEC) strains were more common than any of the other enteropathogens detected by the individual platforms (55 of 95 [57.9%] for FilmArray vs 62 of 134 [46.3%] for Seegene). DEC detection was comparable between the 2 platforms (*P* = .33) (Tables 1 and 2).

**Table 1.** Individual Enteropathogens Detected by the Seegene and FilmArray Platforms

Enteropathogen	Samples by PCR Testing Platform, No. (%)^a^	
	Seegene (Reference Standard; n = 59)	FilmArray (NP-FE Test; n = 60)
EAEC	32 (54.2)	22 (36.7)
*Cryptosporidium* spp	31 (52.5)	25 (41.7)
*Blastocystis hominis*	30 (51)	NA
EPEC	27 (45.8)	24 (40)
*Campylobacter* spp	17 (28.8)	6 (10)
Norovirus	14 (23.7)	1 (1.7)
*Dientameba fragilis*	8 (13.6)	NA
*Clostridioides difficile*	6 (10.2)	2 (3.3)
STEC	3 (5.1)	9 (15)
Sapovirus	2 (3.4)	2 (3.3)
*Aeromonas* spp	2 (3.4)	NA
*Giardia duodenalis*	1 (1.7)	1 (1.7)
*E coli* O157	1 (1.7)	1 (1.7)
Invalid test result	1 (1.7)	0
*Salmonella* spp	0	1 (1.7)
*Entameba histolytica*	0	1 (1.7)
Total individual enteropathogens	134	95

Abbreviations: EAEC, enteroaggregative *Escherichia coli*; EPEC, enteropathogenic *E coli*; NA, not applicable; NP-FE, near-patient field-expedient; PCR, polymerase chain reaction; STEC, Shiga toxin–producing *E coli*.

^a^Enteropathogens are listed in order of frequency of detection by the reference standard Seegene platform; totals in the column headings represent the number of valid tests for the respective PCR platform. NA indicates that the target cannot be detected by the FilmArray platform although it was detectable by the Seegene. The FilmArray has targets not included in the Seegene assays, and vice versa. One Seegene test was invalid and excluded from the analysis.

**Table 2.** Comparison of Enteropathogens Detected by the Seegene and FilmArray Platforms for 60 Selected Samples

Sample Unique Identifier^a^	Seegene PCR Platform (Reference Standard Test)	FilmArray PCR Platform (POC Test)
Negative at POC testing		
S1	Negative	Negative
S2	Negative	Negative
S3	*Blastocystis hominis*, *Cryptosporidium* spp, EAEC, EPEC, ETEC, norovirus, rotavirus	Negative
S4	*B hominis*, *Cryptosporidium* spp, EAEC, EPEC, norovirus, rotavirus	Negative
S5	*Dientameba fragilis*	Negative
Single enteropathogen at POC testing		
S6	*B hominis*	*Entameba histolytica*
S7	*B hominis, Cryptosporidium spp,* EAEC, EPEC, *Giardia lamblia,* norovirus, rotavirus	*Giardia lamblia*
S8	Negative	STEC
S9	*Campylobacter* spp	*Campylobacter* spp
S10	EPEC	EPEC
S11	*B hominis*, *Campylobacter* spp, *Cryptosporidium* spp, EAEC, EPEC, ETEC	*Campylobacter* spp
S12	*Campylobacter* spp	*Campylobacter* spp
S13	*B hominis*, norovirus, rotavirus	STEC
S14	*B hominis,* EPEC	EPEC
S15	*B. hominis,* EPEC	EPEC
S16	*Cryptosporidium* spp	*Cryptosporidium* spp
S17	Negative	*Cryptosporidium* spp
S18	*Campylobacter* spp, *Cryptosporidium* spp	*Cryptosporidium* spp
S19	*B hominis*, *Campylobacter* spp, *Cryptosporidium* spp, EAEC, EPEC, sapovirus	*Cryptosporidium* spp
S20	*Campylobacter* spp, *Cryptosporidium* spp, EAEC	*Cryptosporidium* spp
S21	*Campylobacter* spp, *Cryptosporidium* spp, *D fragilis*	*Cryptosporidium* spp
S22	*B hominis*, *Campylobacter* spp, *Cryptosporidium* spp, EAEC, EPEC, ETEC	*Cryptosporidium* spp
S23	*Cryptosporidium* spp, *D fragilis*	*Cryptosporidium* spp
S24	*B hominis*, *Cryptosporidium* spp	*Cryptosporidium* spp
S25	*B hominis*, *Campylobacter* spp, *Cryptosporidium* spp, EAEC, EPEC	*Cryptosporidium* spp
2 Enteropathogens at POC testing		
S26	*B hominis*, *Campylobacter* spp, *Cryptosporidium* spp	*Cryptosporidium* spp, *Salmonella* spp
S27	Astrovirus, *B hominis*, *Cryptosporidium* spp	Astrovirus, *Cryptosporidium* spp
S28	EAEC	EAEC, EPEC
S29	*B hominis*, *D fragilis*	ETEC, STEC
S30	*B hominis*, *Cryptosporidium* spp, EAEC, EPEC, spp, norovirus	EAEC, EPEC
S31	*B hominis*, *Clostridioides difficile*, *Cryptosporidium* spp, EAEC, EPEC, ETEC, norovirus, rotavirus	EAEC, ETEC
S32	Negative	EAEC, EPEC
S33	*Campylobacter* spp	*Campylobacter* spp, *Cryptosporidium* spp
S34	EAEC	EAEC, STEC
S35	EPEC	EAEC, EPEC
S36	EPEC, ETEC, STEC,	ETEC, STEC
S37	EPEC, ETEC, *B hominis*	EPEC, ETEC
S38	*B hominis*, *Cryptosporidium* spp	*Cryptosporidium* spp, EPEC
S39	*B hominis*, *C difficile*, *Campylobacter* spp, *Cryptosporidium* spp, *D fragilis*, EAEC, EPEC, ETEC, norovirus, rotavirus	*Campylobacter* spp, EAEC
S40	*Aeromonas* spp, *B hominis*, *Escherichia coli* O157, EAEC, EPEC, norovirus, rotavirus, STEC	EAEC, STEC
S41	Invalid	EPEC, ETEC
S42	*B hominis*, *Cryptosporidium* spp, EAEC, EPEC, ETEC, norovirus, rotavirus	EAEC, EPEC
S43	*Cryptosporidium* spp	*Cryptosporidium* spp, STEC
S44	*B hominis, C difficile*, *Cryptosporidium* spp, *D fragilis*, EPEC, EAEC, norovirus, rotavirus	*Cryptosporidium* spp, EPEC
Multiple enteropathogens at POC testing		
S45	*Cryptosporidium* spp, EAEC, EPEC, ETEC	*Cryptosporidium* spp, EAEC, EPEC, ETEC
S46	Negative	*Cryptosporidium* spp, EPEC, ETEC
S47	*Cryptosporidium* spp, EPEC	*Cryptosporidium* spp, EAEC, EPEC
S48	*B hominis*, *Campylobacter* spp, *Cryptosporidium* spp, EAEC, EPEC	*Cryptosporidium* spp, EAEC, EPEC
S49	*Campylobacter* spp, EAEC, EPEC, ETEC	*Cryptosporidium* spp, EAEC, EPEC, ETEC
S50	Rotavirus	*Cryptosporidium* spp, EAEC, EPEC
S51	*B hominis*, *C difficile*, *Campylobacter* spp, *Cryptosporidium* spp, EAEC, EPEC, ETEC, norovirus, rotavirus	EAEC, EPEC, ETEC, rotavirus
S52	*B hominis*, *Cryptosporidium* spp, *D fragilis*, EAEC, EPEC, ETEC, norovirus, rotavirus, sapovirus	EAEC, ETEC, EPEC, sapovirus
S53	*B hominis*, *C difficile*, EAEC, EPEC, norovirus	*C difficile*, norovirus
S54	*B hominis*, *Campylobacter* spp, EAEC, EPEC, ETEC, rotavirus	EAEC, *E coli* O157, ETEC, STEC, rotavirus, sapovirus
S55	*Cryptosporidium* spp	*C difficile*, *Cryptosporidium* spp, EPEC
S56	Astrovirus, *B hominis*, *Campylobacter* spp, *C difficile*, *Cryptosporidium* spp, EAEC, EPEC, STEC, norovirus, rotavirus	*Campylobacter* spp, *Cryptosporidium* spp, EAEC
S57	*Cryptosporidium* spp, EPEC	Astrovirus *Cryptosporidium* spp, EAEC, EPEC
S58	EAEC, EPEC, ETEC	EAEC, EPEC, ETEC
S59	EAEC, EPEC, ETEC	EAEC, ETEC, STEC
S60	*B hominis*, *Cryptosporidium* spp, *D fragilis*, EAEC, EPEC, ETEC, rotavirus	EAEC, EPEC, ETEC

Abbreviations: EAEC, enteroaggregative *E coli*; EPEC, enteropathogenic *E* coli; ETEC, enterotoxigenic *E coli*; POC, point-of-care; PCR, polymerase chain reaction; STEC, Shiga-like toxin–producing *E coli*.

^a^Samples are grouped according to whether they were negative or had 1, 2, or multiple (≥3) enteropathogens at POC testing and are listed in order of detection by Seegene PCR as the reference test. For ease of interpretation, enteropathogen names produced by the automated Seegene Viewer Software are abbreviated per FilmArray nomenclature. The FilmArray platform has targets not included in the Seegene assays, and vice-versa. One Seegene test (S41) was invalid and was excluded from the analysis.

### Comparison of Sensitivity, Specificity, and Interrater Agreement Between Tests

Sensitivity ranged from noncomputable for 2 enteropathogens up to 100%, and specificity ranged from 74% to 100%. The FilmArray platform was highly sensitive (100% [95% confidence interval, 2.50%–100%]) and specific (100% [93.84%–100%]) when compared against the reference standard Seegene test, with an almost perfect agreement (percentage observed agreement, 100%; κ = 1) for *Giardia duodenalis.* The sensitivity was low for *Campylobacter* spp (35.3% [95% confidence interval, 14.21%–61.67%]). This trend toward low sensitivity and high specificity was similar for *Cryptosporidium* spp and other DEC strains. DEC strains were not found to be of major clinical significance, and supportive management and/or isolation were offered as required [6]. Although highly specific for *Salmonella* and *E histolytica*, sensitivity and agreement were not computable for these enteropathogens. The agreement between tests was moderate for *Cryptosporidium* spp, enteroaggregative *E coli* (EAEC), *Campylobacter* spp, and sapovirus. Contrastingly, the agreement between the 2 PCR platforms ranged from none to slight (κ = 0.01–0.20) for *E coli* O157 and norovirus (Table 3).

**Table 3.** Agreement and Interrater Agreement Between FilmArray and Seegene Polymerase Chain Reaction Tests

Enteropathogen^a^	FilmArray vs Seegene PCR Platform			
	Sensitivity (95% CI), %	Specificity (95% CI), %	POA, %	Cohen κ Coefficient^b^
EAEC	63 (42.37–80.6)	84.4 (67.21–94.72)	74.6	0.48
*Cryptosporidium* spp	65 (45.37–80.77)	82.1 (63.11–93.94)	72.9	0.46
EPEC	53.1 (34.74–70.91)	74 (53.72–88.89)	62.7	0.27
*Campylobacter* spp	35.3 (14.21–61.67)	100 (91.59–100)	81.4	0.44
Norovirus	7.14 (0.18–33.87)	100 (92.13–100.00)	77.9	0.11
*Clostridioides difficile*	16.67 (0.42–64.12)	98.11 (89.93 to 99.95)	89.8	0.21
STEC	66.7 (9.43–99.16)	87.5 (75.93–94.82)	86.4	0.28
Sapovirus	50 (1.26–98.74)	98.25 (90.61–99.96)	96.6	0.48
*Giardia duodenalis*	100 (2.50–100)	100 93.84–100	100	1
*Escherichia coli* O157	0 (0–97.50)	98.28 (90.76–99.96)	96.6	−0.02
*Salmonella* spp	NC	98.31 (90.91–99.96)	NC	0
*Entameba histolytica*	NC	98.31 (90.91–99.96)	NC	0

Abbreviations: EAEC, enteroaggregative *E coli*; EPEC, enteropathogenic *E coli*; NC, not computable; PCR, polymerase chain reaction; POA, percentage observed agreement; STEC, Shiga-like toxin–producing *E coli*.

^a^Enteropathogens are listed in order of the frequency of detection with the reference standard Seegene platform.

^b^Cohen's κ coefficients were interpreted as follows: ≤0, no agreement; 0.01–0.20, none to slight agreement; 0.21–0.40, fair agreement; 0.41– 0.60, moderate agreement; 0.61–0.80, substantial agreement; and 0.81–1.00, almost perfect agreement.

## DISCUSSION

This study demonstrates moderate concordance between the NP-FE FilmArray and reference standard Seegene when testing for 5 GI enteropathogens. Concordance was poor to fair for 7 other enteropathogens. FilmArray PCR proved to be highly adaptable as an NP-FE test in a resource-limited environment, and we were able to confirm this by omitting the fecal preservation by Cary Blair medium step. Corresponding samples repatriated in the cold chain had also been stored without Cary Blair preservative before later testing with Seegene PCR. There was no notable impact on the positivity rate, and we found the FilmArray platform to measure comparably in this regard against the selected reference standard Seegene platform, which is already in wide use in clinical microbiological settings as a tool for diarrhea diagnostics [17]. This confirms the versatility of the FilmArray as a suitable platform for use in resource-limited settings, including military deployments and similar environments with challenging logistic chains.

Some previous work challenges the widespread use of multiplex approaches like the FilmArray platform for TD diagnostics, given that in their case only a few enteropathogens (DEC strains) were related to symptoms of TD [19]. Based on our group’s previous experience in close-knit resource-limited settings, as reported elsewhere [6], we would argue that such platforms with a rapid turnaround are crucial for disease containment, particularly in outbreak scenarios. The comprehensive profile of enteropathogens is necessary for accurate epidemiological surveillance, enforcement of force health protection measures, and wider public health measures to support antimicrobial stewardship efforts. All this is critical for maintenance of military operational effectiveness.

### Comparison of Enteropathogen Target Detection

Our data show high specificity for *Campylobacter* spp, *Cryptosporidium* spp, and EAEC of between 84.4% and 100% when the FilmArray platform was compared against the reference standard Seegene. The significant mismatch in detection rates of *Campylobacter* spp for Seegene versus FilmArray PCR (*P* = .03) may be attributed to the diversity and discrepancies in species coverage within the *Campylobacter* genus [21, 22]. To confirm this, we would need to analyze data held by manufacturers, but to our understanding such data are internal and not for publication. Only 2 positives each were detected by the FilmArray platform for *C difficile* and sapovirus and 1 each of *E coli* O157, *E histolytica*, *Giardia duodenalis,* norovirus, and *Salmonella* spp, which are relatively low ODRs to make any meaningful conclusions on concordance.

Interrater agreement for enteropathogenic *E coli* and Shiga toxin–producing *E coli* was fair due to a relatively high number of FPs for both enteropathogens **(**Supplementary Table 1**)**. Although the agreement between platforms for sapovirus was also moderate, and substantial for *Giardia duodenalis* (sensitivity and specificity both 100%), the low ODRs and subsequently the number of TPs also make it impractical to conclusively evaluate concordance. There were no TPs for *Salmonella* spp or *E histolytica* in the NP-FE. Contrastingly, although there were also no TPs for *E coli* O157, there was 1 FP and, as a result, a high percentage observed agreement (>96%), even though there was no agreement between the 2 platforms for this particular target.

### Analysis of Differences Between Platforms

The high ODR we found for DEC is consistent with previous findings [23], as is the good concordance between platforms in the comparison of bacterial targets in general [19, 24]. One significant discrepancy between the 2 platforms was a 14 times greater detection rate of norovirus for the Seegene platform, for which there are several possible reasons. We postulate that FilmArray platform may have been less sensitive for this particular enteropathogen target. Alternatively, the substantial increase in norovirus detection may represent variations in effects of individual platform chemistry on different molecular targets or differences between the nucleic acid extraction, reverse-transcription/amplification processes or indeed the high prevalence and genetic diversity of norovirus. This viral assay distinguishes between norovirus genotypes 1 and 2, while the FilmArray makes no distinction between those genotypes. The Ct values were generally <32 (data not shown) and therefore fairly strong positives, which would rule out a hypothesis of FPs.

Without further sequencing work, we are unable to confirm the possibility of mutations in the assay sites, or whether there are genetic variations in norovirus lineages circulating in rural Kenya, where the study participants initially presented with TD. It is unlikely that the norovirus detections were clinically relevant; the clinical features of the cases were not typical of norovirus infection at the time of the outbreak [6], suggesting the possibility of overdetection by the Seegene platform. *D fragilis* and *B hominis* were detected in 13.3% and 50% of Seegene samples, respectively. Although both protozoal parasites are endemic in some parts of the world and are included in some fecal PCR assays, positive results for *D fragilis* usually indicate the presence of colonizing flora. There is no consensus regarding clinical significance of *D fragilis* [25] because of the comparable frequency of occurrence in patients with GI symptoms and healthy cohorts alike [26]. However, some studies have shown correlation between infection and GI symptoms [27]. In contrast, evidence overwhelmingly points to *Blastocystis* being a commensal [28]. FilmArray PCR does not detect *Aeromonas* spp, *B hominis*, or *D fragilis,* but all 3 are detectable with Seegene assays. We were therefore unable to consider these enteropathogens in our comparison of agreement and interrater agreement between the 2 platforms.

### FilmArray Performance Characteristics and Suitability for Resource-Limited Settings

FilmArray operation does not require the operator to have enhanced training in molecular diagnostic techniques. DNA extraction from clinical samples for molecular analysis is difficult to perform in resource-limited settings due to cost, time, limitations in resources, the risk of contamination, and in some cases lack of relevant expertise [29, 30]. Other advantages of the FilmArray platform include minimal hands-on processing time, rapid turnaround time, platform robustness, and versatility. Some studies have reported high sensitivity and specificity for several GI targets but noted some exceptions [17]. Despite the low concordance of FilmArray testing for the majority of enteropathogens, it remains one of a limited number of closed extraction multiplex platforms on the market suitable as an NP-FE diagnostic tool in resource-limited settings; an additional advantage of this syndromic platform is the compatibility that allows other infectious disease assays to be used within the same platform.

### Study Limitations

Because we were not able to include case controls in this study due to the cost and time constraints alluded to earlier, the attributable fraction could not be evaluated. Our quota sampling may have introduced selection bias, including the possibility of preselecting FP-testing samples. The possibility of reference standard bias must also be considered as the performance characteristics of the Seegene platform may have skewed the perceived accuracy of the FilmArray platform, and we did not compare these multiplex platforms with classic culture-based microbiological analyses. There was 1 sample each with *E coli* O157, *E histolytica, Giardia duodenalis,* norovirus, and *Salmonella* spp, all detected with the FilmArray platform. These smaller individual enteropathogen numbers, as shown in Table 2, may mean that our study was underpowered by the low number of samples, which could have lowered the probability of producing true effects because of the higher chance of type I or II errors (FPs or FNs) [31, 32]. This could likely have produced the skewed findings reported for some enteropathogens (Table 3).

Samples were also analyzed with Seegene PCR 16–18 months after the initial Film Array analysis, with an additional repatriation step from Kenya to the United Kingdom. Although the samples were transported on dry ice and subsequently stored at −80°C, ideally samples from the same aliquots should have been tested across the 2 platforms at the same time points. Likewise, it is not possible to deduce whether or not fecal DNA recovery was impacted due to differing storage and testing steps for both platforms. Data on optimum storage duration of fecal samples over prolonged periods, including quality assurance details on the stability of nucleic acids, are inconsistent [13, 14, 17].

This limitation may account for discrepancies in sensitivity, including overall concordance, and future studies should attempt to address this. Finally, in multinational deployments, complications resulting from lack of standardization may arise from varied standard operating procedures. In our experience, a diagnostic test can only be marked *Conformité Européenne* if the entire workflow from extraction to amplification follows the manufacturer's validated instructions. As far as we are aware, clearance of the Seegene assays for in vitro diagnostic use does not cover nucleic acid extraction with the Qiagen columns. We have already highlighted that this approach likely increased sensitivity for some enteropathogens compared with the nucleic acid extraction schemes covered by the in vitro diagnostic clearance of other available commercial products. Such diverse approaches further complicate collaboration between countries that may follow different regulations.

### Study Strengths

Although the 5 negative samples tested near the point-of care were not true case controls, they were used in addition to ≥10 more samples collected from asymptomatic individuals as quality controls that were run after a predetermined number of FilmArray testing cycles to rule out the risk of cross contamination. We used corresponding samples from a homogenous group within the same resource-limited setting, thereby ruling out the risk of introducing geographic confounders.

### Suggested Future Studies

Expert consensus guidelines should include recommendations for a suitable reference standard multiplex PCR platform for TD diagnostics in resource-limited settings. For qualitative syndromic platforms such as the FilmArray, evidence from case-control studies with larger sample sizes using a combination of clinical and epidemiological data, qualitative as well as quantitative PCR results, including traditional culture-based test outcomes, should be used to help in formulating guidelines for the interpretation of test results to inform treatment decisions and force health protection measures. This is vital for optimizing TD diagnostics and treatment. Such data may also be useful in informing guidelines on distinguishing between commensality or pathogenicity, particularly where multiple enteropathogens are detected. Such a strategy could affect outcomes, including underperformance resulting from morbidity, as well as duty days lost due to bedding down and inappropriate antibiotic use, all which have an overall impact on operational effectiveness.

### Conclusions

Our findings show moderate concordance of FilmArray with Seegene PCR testing in the detection of *Campylobacter* spp, *Cryptosporidium* spp, EAEC, and sapovirus and poor to fair concordance for 9 other enteropathogens. It was impractical to conclusively evaluate concordance and interrater agreement for some enteropathogens due to low ODRs for the NP-FE test, which led to unreliable results. FilmArray test guidelines require refinement due to the test’s inherent limitations, such as low sensitivity for some key enteropathogens. Future interpretation of results to inform clinical practice should be based on robust evidence from large-scale studies, integrating clinical, qualitative, quantitative, and traditional culture data. Despite these shortcomings and in the absence of a reference standard platform, the performance characteristics of FilmArray PCR demonstrated its continued suitability as an NP-FE platform for diarrhea diagnostics in resource-limited settings.

## Supplementary Material

ofag076_Supplementary_Data

## Supplementary Data

Supplementary materials are available at *Open Forum Infectious Diseases* online. Consisting of data provided by the authors to benefit the reader, the posted materials are not copyedited and are the sole responsibility of the authors, so questions or comments should be addressed to the corresponding author.
