# Supplementary material for: Syndromic Diagnostics for Travelers’ Diarrhea: Near-Patient Field-Expedient Testing in Resource-Limited Settings
Source: Open Forum Infect Dis. 2026 Feb 17;13(3):ofag076. doi: 10.1093/ofid/ofag076 (PMC12980125; doi:10.1093/ofid/ofag076)
Supplement: ofag076_Supplementary_Data [file ofag076_supplementary_data.zip › Supplemental Data Sheet 4. DNA extraction .docx]

**Supplemental Data Sheet 4. DNA extraction from fecal samples (QIAamp® PowerFecal^®^ DNA Kit).** All centrifugation steps were performed at room temperature (15–25°C) and the two points below were noted:

- If Solution C1 has precipitated, heat at 60°C until precipitate dissolves.
- Shake to mix Solution C4 before use.

Procedure

1. 0.25 g of stool was added to the Bead Tube provided.

2. 750 μl of PowerBead Solution were added to the Bead Tube.

3. 60 μl of Solution C1 were added and the Bead Tube inverted several times.

4. The tubes were heated at 65°C for 10 min.

5. The tubes were secured horizontally using a Vortex Adapter (cat. no. 13000–V1–24, and vortexed at maximum speed for 10 mins.

6. The tubes were centrifuged at 13,000 x g for 1 min.

7. The supernatant was transferred to the provided 2 ml Collection Tube.

8. 250 μl of Solution C2 was added and vortexed briefly, then incubated at 2–8°C for 5 mins.

9. The tubes were centrifuged at 13,000 x g for 1 min.

10. 600 μl of supernatant were transferred to a clean 2 ml Collection Tube.

11. 200 μl of Solution C3 were added and vortexed briefly, then incubated at 2–8°C for 5 min.

12. The tubes were centrifuged at 13,000 x g for 1 min.

13. The supernatant was transferred to the provided 2 ml Collection Tube.

14. 1200 μl of Solution C4 was added to the supernatant and vortexed for 5 s.

15. 650 μl of supernatant was loaded onto an MB Spin Column and centrifuged at 13,000 x g for 1 min. The flow-through was discarded and this repeated until all the supernatant had been processed.

16. 500 μl of Solution C5 were added and centrifuged for 1 min at 13,000 x g.

17. The flow-through was discarded and the tubes centrifuged again for 1 min at 13,000 x g.

18. The MB Spin Column was placed in the provided 2 ml Collection Tube.

19. 100 μl of Solution C6 was added to the centre of the white filter membrane.

20. The tubes were centrifuged at 13,000 x g for 1 min and the MB Spin Column discarded.

(QIAGEN, Hilden, Gemany).
