# Supplementary material for: Syndromic Diagnostics for Travelers’ Diarrhea: Near-Patient Field-Expedient Testing in Resource-Limited Settings
Source: Open Forum Infect Dis. 2026 Feb 17;13(3):ofag076. doi: 10.1093/ofid/ofag076 (PMC12980125; doi:10.1093/ofid/ofag076)
Supplement: ofag076_Supplementary_Data [file ofag076_supplementary_data.zip › Supplemental Data Table 1. Outcome matrices .docx]

**Supplemental Data Table 1. Outcome matrices for individual enteropathogen comparisons for the FilmArray^®^ and Seegene^™^ tests.**

Enteropathogens are listed in order of frequency of detection by the reference standard Seegene**™**. TP = true positive; TN = true negative; FP = false positive; FN = false negative. NC = not computable. Enteropathogenic *E. coli* = EPEC; Enteroaggregative *E. coli* = EAEC; *Shiga*-like toxin producing = STEC; *Clostridioides difficile* = *C. difficile*; *Entamoeba histolytica* = *E. histolytica.*

| **Enteropathogen** | **FilmArray^®^**  **versus**  **Seegene^™^** | | | | | |
| --- | --- | --- | --- | --- | --- | --- |
|  | **TP** | **TN** | **FP** | **FN** | **Sensitivity (%)**  **95% CI** | **Specificity (%)**  **95% CI** |
| *Cryptosporidium* spp. | 20 | 23 | 5 | 11 | 65  (45.37 - 80.77) | 82.1  (63.11 - 93.94) |
| EPEC | 17 | 20 | 7 | 15 | 53.1  (34.74 - 70.91) | 74  (53.72 - 88.89) |
| EAEC | 17 | 27 | 5 | 10 | 63  (42.37 - 80.6) | 84.4  (67.21 - 94.72) |
| STEC | 2 | 49 | 7 | 1 | 66.7  (9.43 - 99.16) | 87.5  (75.93 - 94.82) |
| *Campylobacter* spp. | 6 | 42 | 0 | 11 | 35.3  (14.21 - 61.67) | 100  (91.59 - 100) |
| *C. difficile* | 1 | 52 | 1 | 5 | 16.67  (0.42 - 64.12) | 98.11  (89.93 to 99.95) |
| Sapovirus | 1 | 56 | 1 | 1 | 50  (1.26 - 98.74) | 98.25  (90.61 - 99.96) |
| *Salmonella* spp. | 0 | 58 | 1 | 0 | NC | 98.31  (90.91 - 99.96) |
| Norovirus | 1 | 45 | 0 | 13 | 7.14  (0.18 - 33.87) | 100  (92.13 - 100.00) |
| *E. histolytica* | 0 | 58 | 1 | 0 | NC | 98.31  (90.91 - 99.96) |
| *Giardia duodenalis* | 1 | 58 | 0 | 0 | 100  (2.50 - 100) | 100  93.84 - 100 |
| *E. coli* O157 | 0 | 57 | 1 | 1 | 0  (0 - 97.50) | 98.28  (90.76 - 99.96) |
