# Supplementary material for: Syndromic Diagnostics for Travelers’ Diarrhea: Near-Patient Field-Expedient Testing in Resource-Limited Settings
Source: Open Forum Infect Dis. 2026 Feb 17;13(3):ofag076. doi: 10.1093/ofid/ofag076 (PMC12980125; doi:10.1093/ofid/ofag076)
Supplement: ofag076_Supplementary_Data [file ofag076_supplementary_data.zip › renamed_2ddc5.docx]

**Supplemental Data Sheet 1. Seegene^™^, Allplex^™^ GI Panel Assays and targets**

The 25 enteropathogen targets for Seegene^™^, Allplex^™^ directly from fecal specimens. *E. coli* = *Escherichia coli*.

| **Allplex^™^ GI-Bacteria(I) Assay** | **Allplex^™^ GI-Bacteria (II) Assay** |
| --- | --- |
| - *Aeromonas spp.* (Aer) - *Campylobacter spp.* (Cam) - *Clostridioides difficile* toxin B (CdB) - *Salmonella spp.* (Sal) - *Shigella spp./EIEC* (Sh/EI) - *Vibrio spp.* (Vib) - *Yersinia enterocolitica* (Yer) | - Enteroaggregative *E. coli* [EAEC] (aggR) - Enteropathogenic *E. coli* [EPEC] *(eaeA)* - *E. coli* O157 (*E. coli* O157) - Enterotoxigenic *E. coli* [ETEC] *(lt/st)* - Hypervirulent *Clostridioides difficile* (CD hyper) - Shiga-like toxin-producing *E. coli* [STEC] (stx1/2) |
| **Viruses** | **Parasites** |
| - Adenovirus (AdV) - Astrovirus (AstV) - Norovirus GI (NoV-GI) - Norovirus GII (NoV-GII) - Rotavirus (RotV) - Sapovirus (SV) | - *Blastocystis hominis* (BH) - *Cryptosporidium* spp*.* (CR) - *Cyclospora cayetanensis* (CC) - *Dientamoeba fragilis* (DF) - *Entamoeba histolytica (EH)* - *Giardia lamblia (duodenalis)* (GL) |

Reference:

Allplex^™^Gastrointestinal Panel Assays (Seoul, South Korea). Available from: https://www.seegene.com/assays/allplex_gastrointestinal_panel_assays
