# Supplementary material for: Syndromic Diagnostics for Travelers’ Diarrhea: Near-Patient Field-Expedient Testing in Resource-Limited Settings
Source: Open Forum Infect Dis. 2026 Feb 17;13(3):ofag076. doi: 10.1093/ofid/ofag076 (PMC12980125; doi:10.1093/ofid/ofag076)
Supplement: ofag076_Supplementary_Data [file ofag076_supplementary_data.zip › renamed_76a0e.docx]

**Supplemental Data Sheet 3: BioFire^™^ FilmArray^™^ PCR testing protocol**

1. Place the FilmArray^®^ pouch into the loading block and inject the hydration solution into the pouch through the blue inlet port on the right.
2. Add approximately 0.2ml of sample into the sample buffer, mix the solution then inject it into the pouch through the red inlet port on the left.
3. Load the FilmArray^®^ pouch into the FilmArray^®^ instrument and scan the pouch ID using the barcode reader.
4. Finally, enter any unique sample identifiers and start the automated run which completes by producing a test report.

(bioMérieux, Marcy-l'Étoile, France).
