# Supplementary material for: Syndromic Diagnostics for Travelers’ Diarrhea: Near-Patient Field-Expedient Testing in Resource-Limited Settings
Source: Open Forum Infect Dis. 2026 Feb 17;13(3):ofag076. doi: 10.1093/ofid/ofag076 (PMC12980125; doi:10.1093/ofid/ofag076)
Supplement: ofag076_Supplementary_Data [file ofag076_supplementary_data.zip › renamed_84f55.docx]

**Supplemental Data Sheet 2.** **PCR BioFire^®^ FilmArray^®^ GI Panel targets**

The 22 enteropathogen targets for PCR BioFire^®^ FilmArray^®^ directly from fecal specimens. *E. coli* = *Escherichia coli*.

| **Bacteria** | **Diarrheagenic *E. coli or Shigella spp.*** |
| --- | --- |
| - *Campylobacter* (*jejuni, coli* & *upsaliensis*) - *Clostridioides difficile* (Toxin A/B) - *Plesiomonas* *shigelloides* - *Salmonella spp.* - *Yersinia enterocolitica* - *Vibrio* (*parahaemolyticus, vulnificus*, & *cholerae*) - *Vibrio cholerae* | - *E. coli* O157 - Enteroaggregative *E. coli* (EAEC) - Enteroinvasive *E. coli* (EIEC) - Enteropathogenic *E.* coli (EPEC) - Enterotoxigenic *E. coli* (ETEC) lt/st - Shiga-like toxin-producing *E. coli* (STEC) stx1/stx2 *E. coli* O157 |
| **Viruses** | **Parasites** |
| - Adenovirus F 40/41 - Astrovirus - Norovirus GI/GII - Rotavirus A - Sapovirus (I, II, IV, and V) | - *Cryptosporidium* - *Cyclospora cayetanensis* - *Entamoeba histolytica* - *Giardia duodenalis* |

Reference:

Buss SN, Leber A, Chapin K, et al. Multicenter evaluation of the BioFire^®^ FilmArray^®^ Gastrointestinal Panel for etiologic diagnosis of infectious gastroenteritis. J Clin Microbiol 2015; 53:915–925. doi:10.1128/JCM.02674-14.
